# The Oxygen Treatment of Ulcers

**Published:** 1897-03-13

**Authors:** 


### The Oxygen Treatment of Ulcers.

We have summarised in another column the many communications which we have received from hospital surgeons on the Oxygen Treatment of Ulcers.

Two very different questions are involved; one as to the facts, the other as to the ethics. As to the first: Is it true that the application of oxygen, in the manner described, is of service in the healing of ulcers, and are the bacteriological changes spoken of by Mr. Stoker found to occur by other observers? As to ethics: Is it ethically correct, even if the oxygen treatment be right and proper in itself, that a special "home" should be established for the exclusive practice of this particular precedure? In regard to the scientific question—the question of fact—it is, we think, a very regrettable circumstance that a treatment which has been put forward so positively, and has been supported by an array of statements in regard to the bacteriology involved equally definite, should not have been so tested in the wards of our general hospitals as to have long since received either confirmation or refutation.

Several among the writers of the communications which we have received appear to have had some general experience of the treatment, and although most of them are quite sceptical as to its utility, some speak well of it. But no one seems to have tested the whole question, including the bacteriology, as a scientific problem, and, much as we doubt the correctness of some of the assumptions on which it is founded, it has to be confessed that to a very large extent the objections raised against the treatment are founded on general experience rather than on exact knowledge. Of course investigators cannot always be going again over old ground; astronomers are not bound to take fresh measurements to prove that the earth is round every time a fanatic reasserts that it is flat, and old experiments need not be repeated to prove the pathogenic activity of certain micro-organisms just because Mr. Stoker says they are beneficial.

But that is not exactly the form the problem takes. The assertion made is, not that they are always beneficial, but that under certain fresh conditions these organisms take on fresh activities; and this is a matter which, as one of our correspondents points out, can only be verified by a bacteriologist working at a hospital where the cases are treated in the manner described, and the organisms grow under the conditions indicated.

When we come to the ethical side of the question, and discuss the propriety of establishing a "home" for the trial of the treatment, the matter is again complicated by the small interest shown in the matter at the general hospitals. If so soon as Mr. Stoker put forward his suggestion some scientific surgeon, among the many in London, had offered to test the treatment for good and all, with all the appliances at his disposal in some large hospital, we should have looked with the most absolute disfavour on the establishment of a special institution of the sort. But things have not so happened, and one reason of this is that chronic ulcers are not generally taken into the wards of the hospitals, one of our correspondents saying "we do not care to give up beds for the cure of chronic ulcers," and "we are glad to get rid of them at any price."

Several of our correspondent agree that the treatment suggested onght to be definitely tested in the hospitals, and almost all say that there is no necessity whatever for the establishment of a special hospital for the purpose. The first thing then is that the matter should be tested. Let it be put to trial and either accepted or rejected; but, at any rate, let us get rid of the present anomalous condition of affairs. Special hospitals are bad enough, but special institutions for particular lines of treatment are far worse, in that the patient in choosing his institute chooses at the same time the treatment he will have performed upon him. If it is true that certain diseases are tabooed at the general hospitals, by all means let us have special ones for their reception. However undesirable, that is at least legitimate. But in the name of all that is reasonable, do not let us drift into a system of Carbolic Hospitals, and Sublimate Institutes, and Oxygen Homes.

